# Endothelin receptors in renal interstitial cells do not contribute to the development of fibrosis during experimental kidney disease

**DOI:** 10.1007/s00424-021-02604-4

**Published:** 2021-08-06

**Authors:** Thomas H. Neder, Julia Schrankl, Michaela A. A. Fuchs, Katharina A. E. Broeker, Charlotte Wagner

**Affiliations:** Institute of Physiology, University of Regensburg, Universitätsstraße 31, D-93053 Regensburg, Germany

**Keywords:** Endothelin-1, Endothelin receptors, Kidney fibrosis, Unilateral ureter occlusion, Adenine-induced nephropathy

## Abstract

Renal interstitial fibrosis is characterized by the development of myofibroblasts, originating from resident renal and immigrating cells. Myofibroblast formation and extracellular matrix production during kidney damage are triggered by various factors. Among these, endothelins have been discussed as potential modulators of renal fibrosis. Utilizing mouse models of adenine nephropathy (AN) and unilateral ureter occlusion (UUO), this study aimed to investigate the contribution of endothelin signaling in stromal mesenchymal resident renal interstitial cells. We found in controls that adenine feeding and UUO caused marked upregulations of endothelin-1 (ET-1) gene expression in endothelial and in tubular cells and a strong upregulation of ET_A_-receptor (ET_A_-R) gene expression in interstitial and mesangial cells, while the gene expression of ET_B_-receptor (ET_B_-R) did not change. Conditional deletion of ET_A_-R and ET_B_-R gene expression in the FoxD1 stromal cell compartment which includes interstitial cells significantly reduced renal ET_A_-R gene expression and moderately lowered renal ET_B_-R gene expression. ET receptor (ET-R) deletion exerted no apparent effects on kidney development nor on kidney function. Adenine feeding and UUO led to similar increases in profibrotic and proinflammatory gene expression in control as well as in ET_A_^flfl^ET_B_^flfl^ FoxD1^Cre+^ mice (ET-Ko). In summary, our findings suggest that adenine feeding and UUO activate endothelin signaling in interstitial cells which is due to upregulated ET_A_-R expression and enhanced renal ET-1 production Our data also suggest that the activation of endothelin signaling in interstitial cells has less impact for the development of experimentally induced fibrosis.

**Supplementary Information:**

The online version contains supplementary material available at 10.1007/s00424-021-02604-4.

## Introduction

Development and progression of renal fibrosis is a characteristic of chronic kidney disease and is widely believed as the consequence of an excess accumulation of extracellular matrix (ECM) proteins such as collagens, fibronectin, or tenascins [7, 27, 46, 55]. Progressive fibrosis results in deterioration of tubular and glomerular function [63]. It is well established that myofibroblasts are the key mediators of fibrosis by serving as the primary matrix/collagen-producing cells. These myofibroblasts transdifferentiate from several cell types including fibroblasts, pericytes, monocytes, tubular, and endothelial [6, 15, 18, 20, 28, 31, 41, 64]. There is broad agreement that fibroblasts, pericytes, and bone marrow–derived cells contribute equally to the myofibroblast population [6, 15, 32, 37, 39]. Resident fibroblasts and pericytes derive from the FoxD1^+^ stroma progenitor cell population, and they express the platelet-derived growth factor receptor β (PDGFR-β) [37]. A recent study showed that in human kidneys mainly PDGFR-β cells undergo transformation into myofibroblasts [37].

Among a variety of cytokines and signaling factors involved in myofibroblast formation and the progression of fibrosis, the role of ET-1 has been studied in various experimental models [2, 5, 45, 48]. ET-1 binds to either ET_A_- or ET_B_-R which mainly activate the inositol triphosphate signaling cascade intracellularly [26, 50, 52]. In damaged kidneys, an increase of ET-1 and ET_A_-R mRNA expression has been already reported [1, 9, 33, 43, 44, 65]. An important role of ET-1 in renal fibrosis was elucidated from the finding that transgenic mice overexpressing human ET-1 develop renal abnormalities associated with interstitial fibrosis [25] and that inhibitors of endothelin receptors can attenuate experimentally induced fibrosis [5, 45, 48]. Since ET-R are expressed in different cells of the kidney, it is difficult to gain insights into the cell type-specific roles of ET-R by experiments systematically inhibiting the ET-1 signaling pathway. This could explain some of the controversial reports in recent years [2, 5, 35, 45, 48].

In view of the major role of interstitial cells as precursors of myofibroblasts, we were interested to define the role of endothelin signaling in this cell population.

To this aim, we generated a mouse model with constitutive genetic ablation of both ET_A_- and ET_B_-R in cells descending from the stroma progenitor cell population which is characterized by the specific expression of the transcription factor FoxD1. Besides interstitial fibroblasts/pericytes, also vascular smooth muscle cells, renin-producing cells, and mesangial cells derive from the FoxD1^+^ progenitor cell population [40, 57]. ET-Ko mice were studied in two models of experimental renal fibrosis, unilateral ureter occlusion (UUO) and adenine-induced nephropathy (AN). AN is a chronic damage model mediated by precipitation of crystals within the tubular lumen leading to kidney injury similar to that in human crystal-induced pathologies [6, 32, 51]. UUO, on the other hand, represents an acute damage model for mechanical stress [6, 10]. In these pathological models, the effects of ET-R deletion in stromal cells on the expression of α-SMA as a marker for myofibroblast formation were examined. Furthermore, we investigated the profibrotic and proinflammatory gene expression.

## Material and methods

### Animals

ET_A_^flfl^ ET_B_^flfl^ FoxD1^Cre+^ mice were generated by crossbreeding FoxD1^Cre+^mice (JAX stock #029684) and mice with loxP-flanked ET_A_ (obtained from Dr. M. Yanagisawa at the Howard Hughes Institute at University of Texas Southwestern Medical Center) [30] and loxP-flanked ET_B_ alleles (obtained from Dr. M. Epstein, University of Wisconsin, Madison) [14]. Genotyping was performed using the primers listed in Table 1*.* Littermates negative for Cre were used as control animals. Animals were maintained on standard rodent chow (0.6% NaCl; Ssniff, Soest, Germany) with free access to tap water. All animal experiments were performed according to the Guidelines for the Care and Use of Laboratory Animals published by the US National Institutes of Health and approved by the local ethics committee.

**Table 1:** Primer sequences used for genotyping of mice*.*

Genotype	Sequence (5′to 3′), fwd	Sequence rev (5′to 3′), rev
FoxD1^Cre^	gaactgtcaccggcagga	aggcaaattttggtgtacgg
ET_B_ KO	tggaatgtgtgcgaggcc	cagccagaaccacagagaccaccc
ET_B_ wt	ctgaggagagcctgattgtgccac	cgactccaagaagcaacagctcg
ET_A_ flox	gggtggcatttaccaccaga	gcgtagcctcacaagcacat

### Adenine-induced nephropathy

Adenine-induced fibrosis was generated in adult mice for this study [6, 29, 32, 51]. Male mice were fed with adenine containing diet (0.2%) continually for 3 weeks. Experiments were performed after exactly 3 weeks (3-week adenine).

### Unilateral ureteral obstruction

Under inhalation anesthesia, a ureteral ligation was placed close to the right kidney through a small abdominal incision [15]. Mice were kept under close observation after the operation for 72h. Five days after the procedure, mice were killed and perfused for RNAscope, or the kidneys were removed for mRNA quantification.

### In situ hybridization via RNAscope

Localization of mRNA was studied with the RNAscope Multiplex Fluorescent v2 kit (Advanced Cell Diagnostics ACD, Hayward, CA, USA), according to the manufacturer’s instructions. The kidneys were perfusion-fixed with 10% neutral buffered formalin solution, dehydrated in an ethanol series, and embedded in paraffin. Hybridization signals were detected on 5μm tissue sections using the TSA® Plus fluorophores Cy3 and Cy5 (PerkinElmer, Waltham, MA). Slices were mounted with ProLong Gold Antifade Mountant (Thermo Fisher Scientific, Waltham, MA) and viewed with an Axio Observer.Z1 Microscope (Zeiss, Jena, Germany). Positive and negative controls were routinely enclosed. RNAscope® probes are listed in Table 2.

**Table 2:** RNAscope probes used for in situ hybridization

RNAscope® probe	Cat no.
Mm-ET1	435221
Mm-ETA	486351
Mm-ETB	473801
Mm-Pdgfrb-C2	411381-C2
Mm-CD31-C2	316721-C2
Mm-Col1a1	319371
Mm-F4/80	460651
Mm-asma	319531
2.5 Duplex Positive Control Probe-Mm	321651
2-plex Negative Control Probe	320751

### Determination of mRNA expression by real-time PCR

Total RNA was isolated from kidneys as described by Chomczynski and Sacchi [11] and quantified by a photometer. Of the resulting RNA, 1μg was used for reverse transcription. cDNA was synthesized by Moloney murine leukemia virus RT (Thermo Fisher Scientific, Waltham, MA). For quantification of mRNA expression, real-time PCR was performed using a LightCycler Instrument and the LightCycler 480 SYBR Green I Master Kit (Roche Diagnostics, Mannheim, Germany). mRNA expression data were normalized to glyceraldehyde 3-phosphate dehydrogenase (GAPDH). Sequences of the primers for the real-Time PCR are shown in Table 3.

**Table 3:** Primer sequences used for real time PCR

**Genes**	**Sequence (5′-3′)**	**Sequence (3′-5′)**
Col1a1	ctgacgcatggccaagaaga	atacctcgggtttccacgtc
Col3a1	ggtggttttcagttcagctatgg	ctggaaagaagtctgaggaatg
ET1	ccacagaccaggcagttagat	tgaatggtactttgggccctga
ETA	aggaacggcagcttgcggat	agcaacagaggcaggactga
ETB	ggagagcggtatgcagattg	tattgctggaccggaagttg
Fibronectin	tccagccccaccctacaagt	ccagaccaaaccataagaac
Tenascin C	tgaaccacaagaaataaccctc	gttgctatggcactgactgg
CX3CR1	aagttcccttcccatctgct	caaaattctctagatccagttcagg
CX3CL1	cacctcggcatgacgaaat	ttgtccacccgcttctcaa
GAPDH	caccagggctgccatttgca	gctccacccttcaagtgg
α-SMA	actgggacgacatggaaaag	catctccagagtccagcaca

### Immunohistochemistry

For immunoreactivity 5-μm sections of the kidneys fixed in 3% PFA were blocked with 10% horse serum/1% BSA in PBS and were incubated either with rabbit anti ET-1 (ab117757, Abcam, Cambridge, UK), mouse anti-αSMA (ab7817, Abcam, Cambridge, UK), or rabbit anti-col1a1 (ab34710-100, Abcam) in different experimental approaches at 4°C overnight. After washing with BSA/PBS, sections were incubated with Cy3 and Cy5 secondary antibodies (Dianova, Hamburg, Germany), mounted with Glycergel (Agilent, Waldbronn, Germany), and viewed with an Axio Observer.Z1 Microscope.

### Systolic blood pressure measurement

Systolic blood pressure of conscious mice was determined by tail-cuff manometry (TSE Systems). Animals were placed into the holding device for 5 consecutive days before the first measurement. Blood pressure was measured daily for 10 days in a row, and the average of these measurements was used for analysis.

### Determination of glomerular filtration rate

For glomerular filtration rate (GFR) measurement, FITC-labeled sinistrin (3.74 μl/g body wt) was injected retro-orbitally in a single bolus. Approximately 5 μl of blood was collected from the tail vein of conscious mice at 3, 7, 10, 15, 35, 55, and 75 min after injection. After centrifugation, 0.5 μl of the plasma samples were diluted in HEPES (0.5 M, pH 7.4), and FITC fluorescence was measured by Invitrogen Qubit 3.0 Fluorometer (Thermo Fisher Scientific).

### Urine analysis

Urine osmolality was determined by freezing point measurements of the urine samples (Osmomat 030, Gonotec). Urine sodium and potassium concentrations were determined by flame photometer (XP flame photometer; BWB Technologies).

The determination of ET-1 in urine was carried out with an ET-1 ELISA from R&D Systems (Minneapolis, MN, USA) according to the manufacturer’s instructions. Measurements of the urine albumin concentration were determined with an albumin ELISA (ICL, E-90AL, Portland, OR, USA) according to the manufacturer’s instructions.

### Determination of hematocrit values, plasma renin, and plasma erythropoietin concentration

Blood samples were taken from tail vein into EDTA-coated capillary tubes to prevent clotting. Hematocrit values were determined after centrifugation (8 min, 8,000 rpm). The erythropoietin (EPO) concentration was determined in plasma samples using the Quantikine Mouse EPO ELISA kit (R&D Systems, Minneapolis, MN) according to the manufacturer’s protocol. Plasma renin concentration was determined by measuring the capacity of plasma samples to generate ANG I in the presence of excess renin substrate. Therefore, plasma samples were incubated for 90 min at 37°C with plasma from bilaterally nephrectomized male rats. The generated ANGI (in ng·ml^−1^·h^−1^) was determined by ELISA (IBL International, Hamburg) according to manufacturer’s protocol.

### Determination of plasma urea and creatinine concentrations

Plasma urea concentration was determined in plasma samples using the QuantiChrom™ Urea Assay Kit ( Bioassay Systems, CA, USA) according to manufacturer’s protocol. Plasma creatinine concentration was determined by Creatinine Serum Detection Kit (Arbor Assays, MI, USA) according to manufacturer’s protocol.

### Statistical analyses

All data are presented as mean ± SEM. Statistical significance was determined by ANOVA. *p* < 0.05 was considered statistically significant. The data were analyzed using GraphPad Prism8.

## Results

### The endothelin system is activated during experimental kidney disease

#### Endothelin 1

**Fig. 1:**
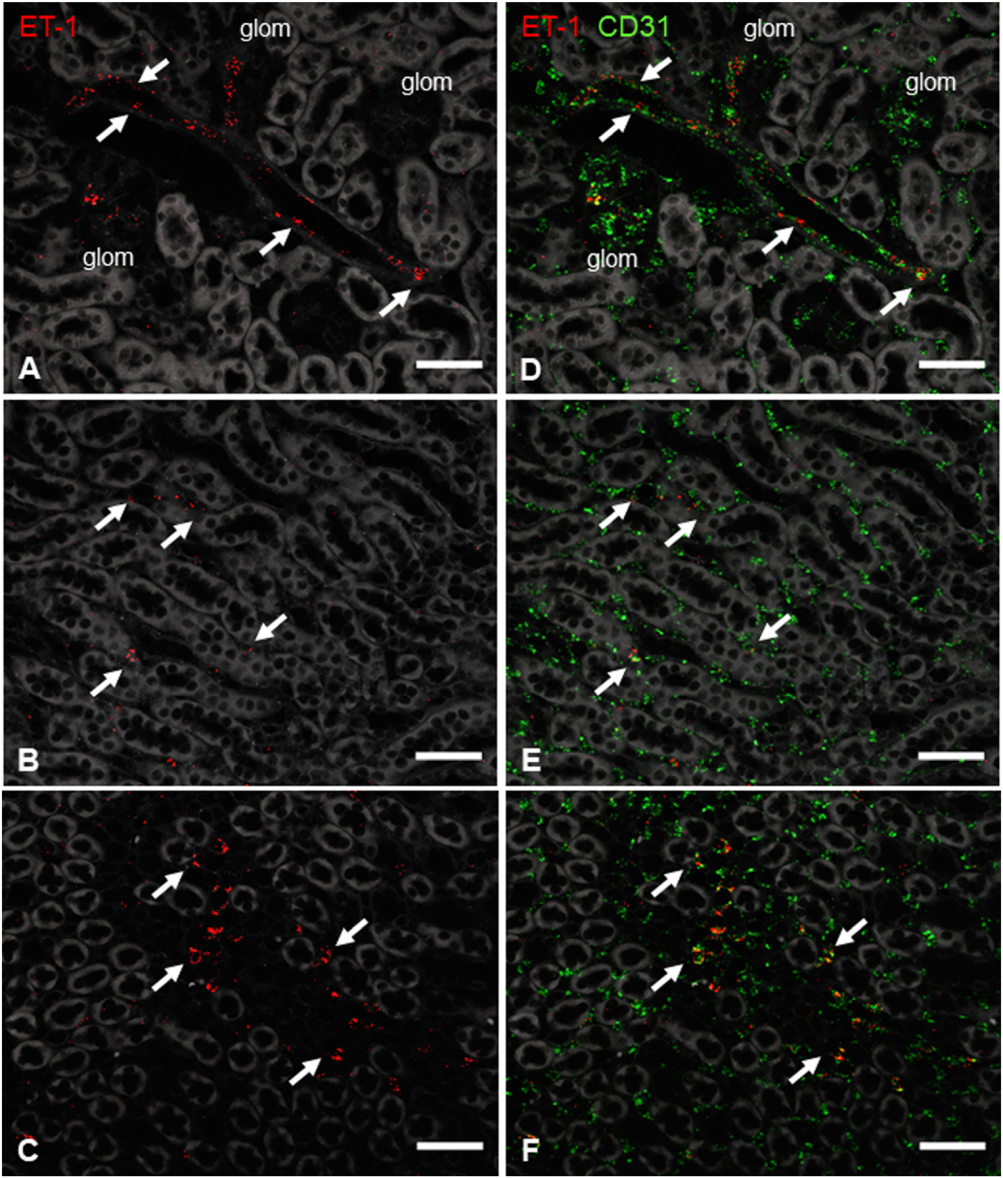
Basal expression of ET-1 mRNA on kidney sections of control mice. Details showing RNAscope for ET-1 mRNA (red) in cortex (**A**), outer (**B**), and inner medulla (**C**) on a control kidney section. ET-1 mRNA was detected within glomeruli (glom) and renal vessels (arrows). Merged details of the co-hybridization of ET-1 with the endothelial marker CD31 (green) (**D**, **E**, **F**) revealed endothelial cells as the only expression site of ET-1 synthesis in the healthy kidney. Scale bars = 50μm

**Fig. 2:**
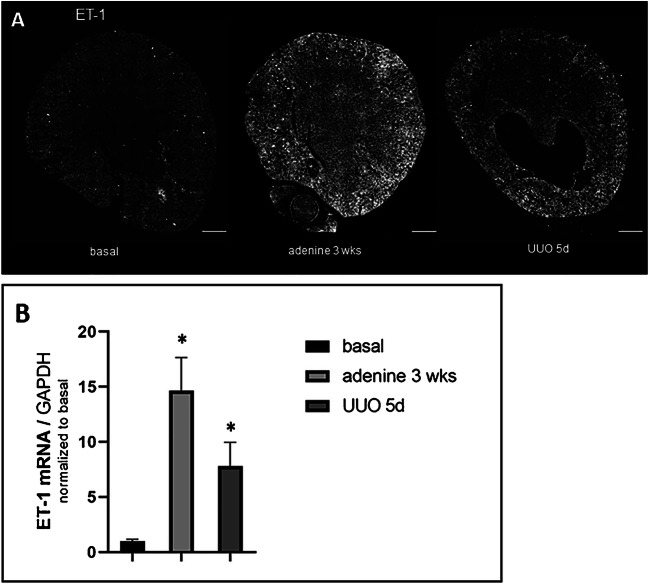
ET-1 mRNA abundance in control mice under basal and pathological conditions. **A** RNAscope for ET-1 mRNA expression on whole kidney sections of control mice under basal conditions, after adenine feeding for 3 weeks and UUO for 5 days. Scale bars = 500μm. **B** ET-1 mRNA abundance of control mice under basal condition, after adenine feeding and after UUO for 5 days. Renal ET-1 mRNA levels increased 15-fold due to adenine nephropathy and 8-fold after UUO for 5 days. All data are means ± SEM of at least 5–8 animals per condition. Single asterisk is p<0.05 compared to untreated animals

**Fig. 3:**
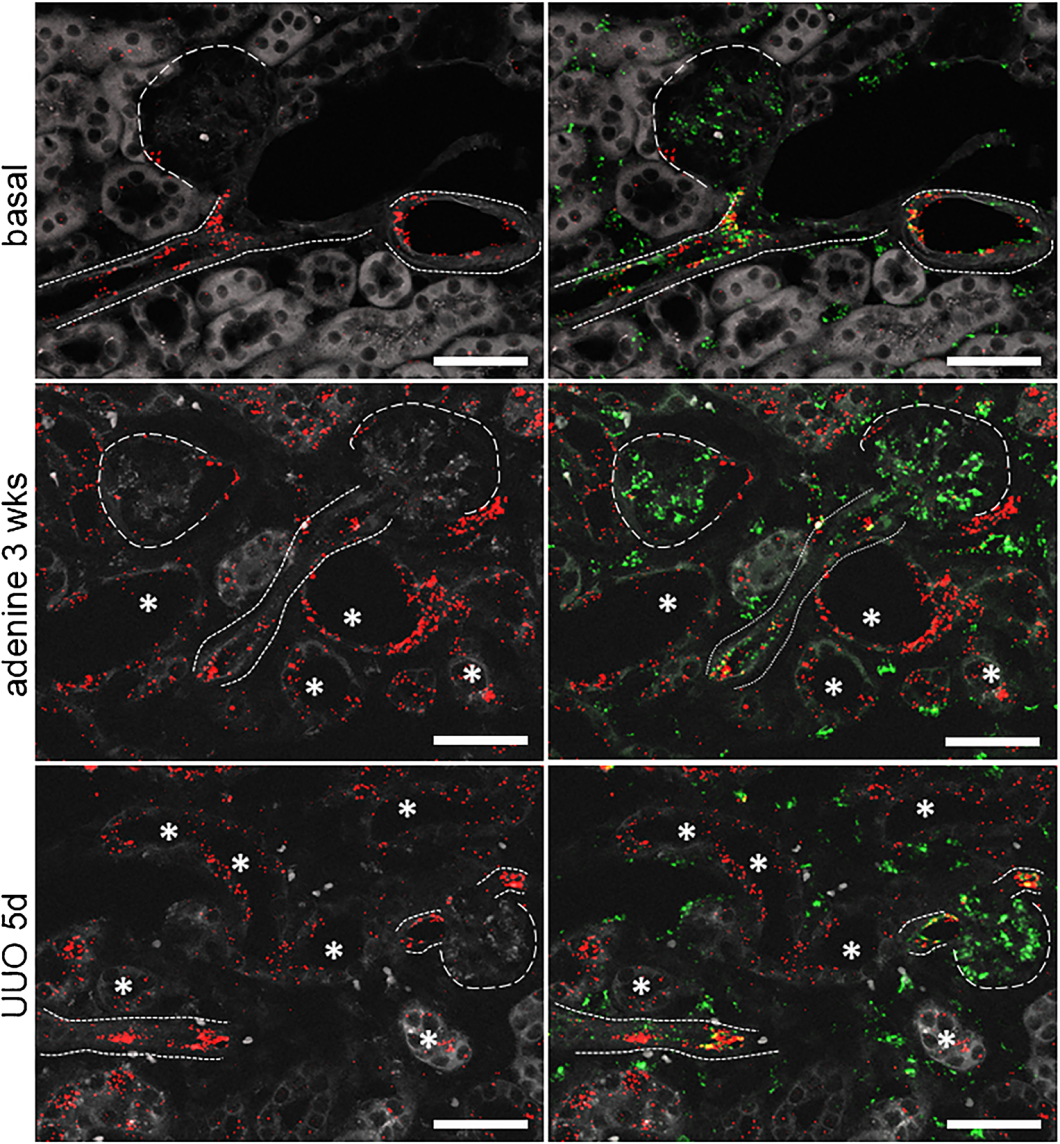
ET-1 mRNA localization in the control kidney under basal and pathological conditions. ET-1 mRNA localization in the mouse kidney under basal conditions (upper panel), after 3 weeks of adenine feeding (middle panel) and 5-day UUO (lower panel). Asterisks indicate ET-1 expression (red) in tubular segments, the finely dotted line indicates ET-1 within renal vessels, the coarser dashed line surrounds glomeruli. Co-hybridization with the endothelial marker CD31 (green) revealed ET-1 production in endothelial cells of glomeruli, in the endothelium of renal vessels and peritubular capillaries. Scale bars = 50μm

ET-1 mRNA expression was localized by RNAscope on kidney sections of healthy control mice. ET-1 mRNA was detected in endothelial cells of glomeruli and endothelial cells lining intrarenal blood vessels (Fig.1). ET-1 mRNA hybridization signals were the strongest in the cortical zone but more faint in the outer and inner medulla where they appear in the endothelium of capillary renal vessels (Fig 1). Adenine feeding for 3 weeks and unilateral ureter occlusion (UUO) for 5 days led to a 15- and 8-fold increase in ET-1 mRNA abundance, respectively (Fig. 2). Upregulated ET-1 expression was observed in endothelial cells and de novo in tubular cells (Fig. 3).

#### ET receptors

**Fig. 4:**
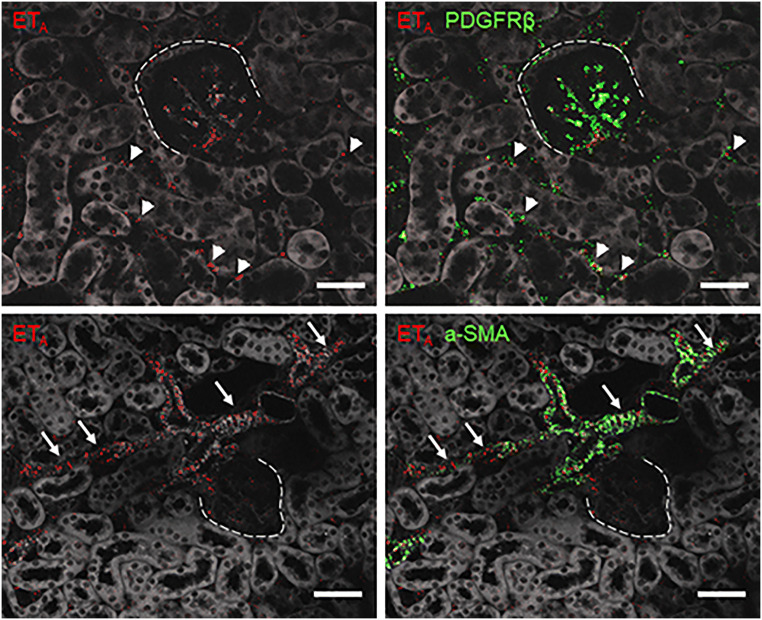
Details of an RNAscope for ET_A_ mRNA localization on kidney sections of control mice. RNAscope for ET_A_ (red) with co-hybridization of the mesenchymal cell marker PDGFR-β (green) in the upper panel showed ETA in mesangial cells within glomeruli (dotted line) and in renal interstitial cells (arrowheads). ET_A_ mRNA is also synthesized in vascular smooth muscle cells of renal vessels (arrows), clarified by a-SMA co-localization. Scale bars = 50μm

**Fig. 5:**
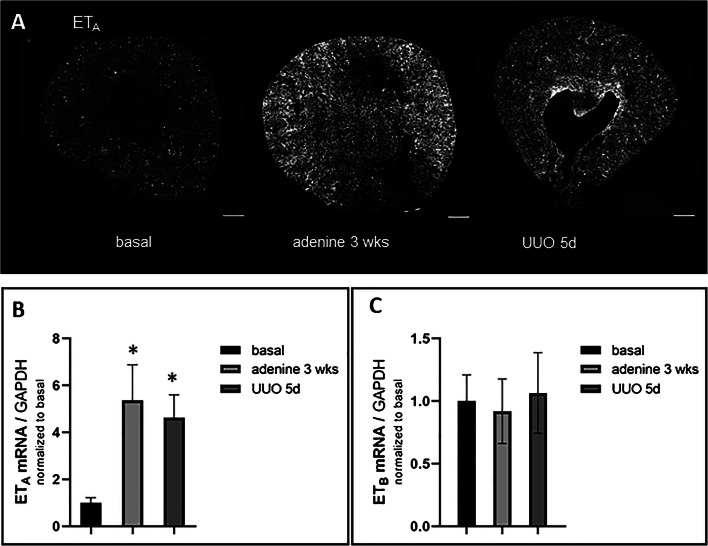
ET receptor mRNA abundance in control mice under basal and pathological conditions. **A** RNAscope for ET_A_ mRNA expression on whole kidney sections of control mice under basal conditions, after adenine feeding for 3 weeks and UUO for 5 days. Scale bars = 500μm. **B** ET_A_ mRNA abundance of untreated mice (basal), after adenine feeding and after UUO for 5 days. Renal ET_A_ mRNA levels increased about 5-fold in both experimental models. **C** ET_B_ mRNA abundance did not change in the pathological models. All data are means ± SEM of 5–8 animals per condition. Single asterisk is *p*<0,05 compared to untreated animals. Note the different scales between data for ET_A_ and ET_B_ mRNAs

RNAscope for ET_A_ mRNA showed clear hybridization signals in vascular smooth muscle cells, mesangial cells, and mesenchymal interstitial cells of the healthy kidney (Fig. 4). The latter two cell types could be identified by co-hybridization with PDGFR-β (Fig. 4, upper panel) while ET_A_-R expression in vascular muscle cells was confirmed by co-hybridization with α-SMA (Fig.4, lower panel). All ET_A_-R mRNA expressing cells have their origin in the FoxD1-positive stroma precursor compartment. During adenine feeding and UUO, ET_A_ mRNA expression increased about 5-fold (Fig. 5A, B), whereas ET_B_-R mRNA remained unchanged (Fig. 5C).

**Fig. 6:**
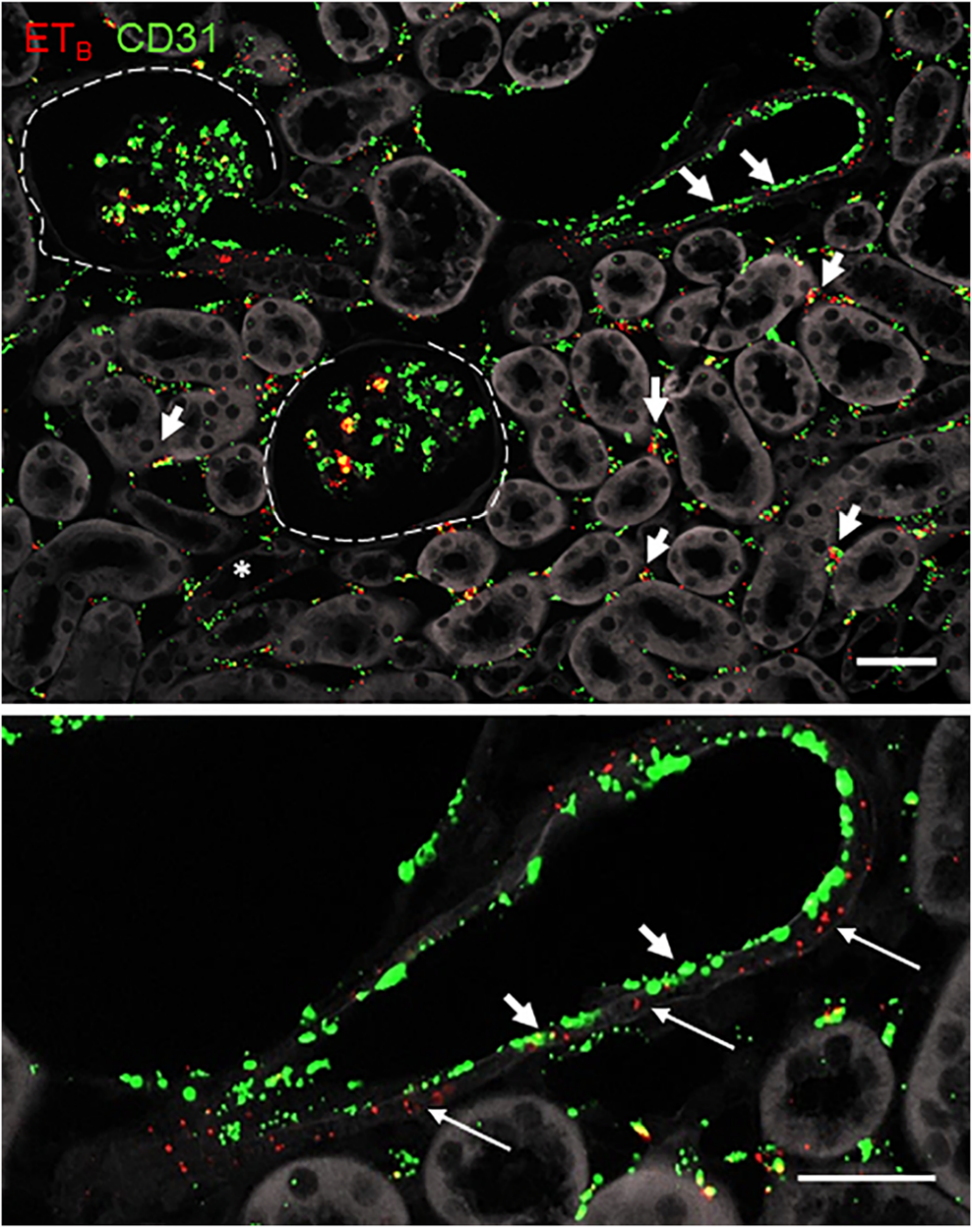
Details of an RNAscope for ET_B_ and CD31 mRNA on kidney sections of a control mouse. RNAscope for ET_B_ (red) and CD31 (green) on kidney sections of control mice under basal conditions in the cortex. (upper panel). Asterisks indicate expression in tubular segments, arrowheads ET_B_ expression in endothelial cells. Scale bar = 50μm. The detailed view (lower panel) shows ETB expression in vascular smooth muscle cells. Arrows indicate expression in vascular smooth muscle cells, arrowheads in the endothelial layer. Scale bar = 50µm

Co-RNAscope for ET_B_-R mRNA and the endothelial marker CD31 showed expression of ET_B_-R in glomerular, perivascular, and vascular endothelial cells (Fig. 6). In addition, ET_B_-R mRNA was found in different tubular segments (Fig. 6) and also showed weak expression in the medial layer of renal vessels ( Fig. 6, lower panel).

**Fig. 7:**
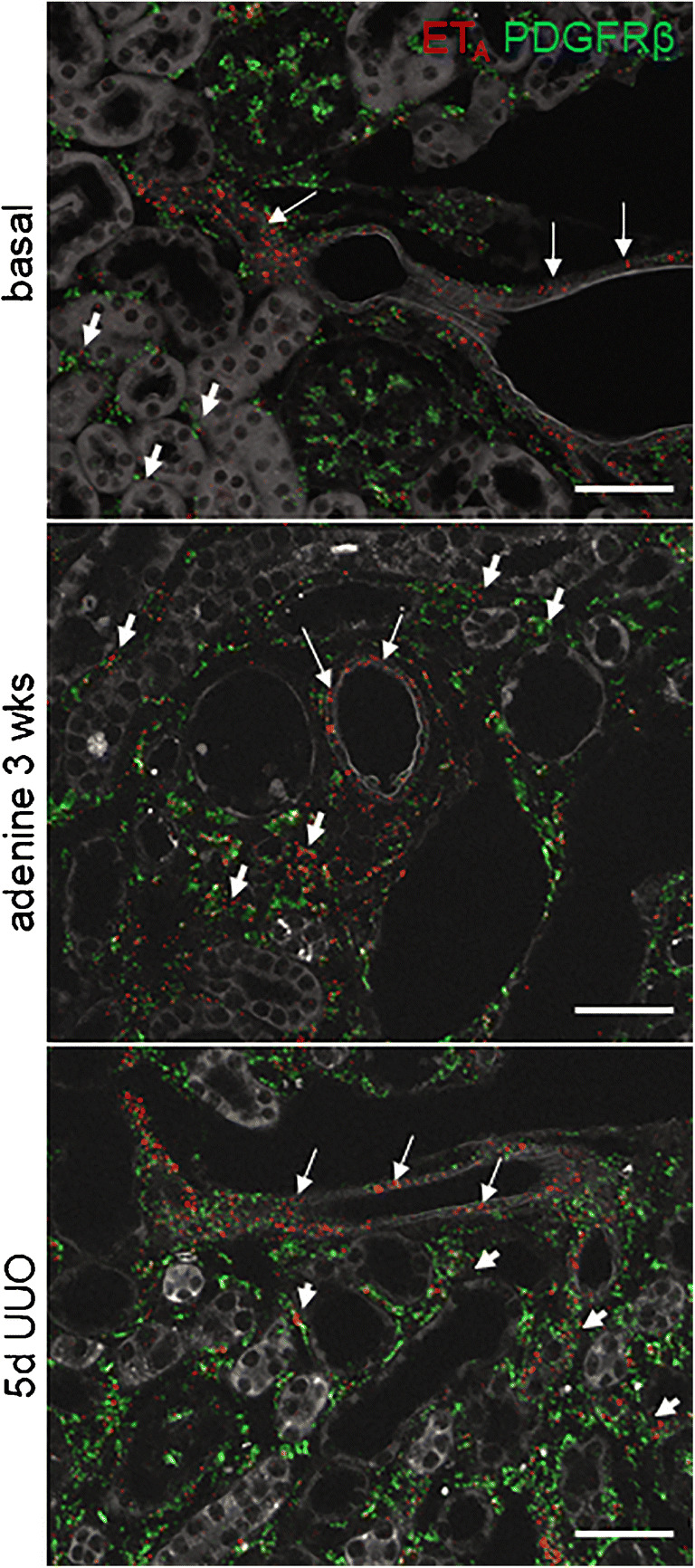
Expression of ET_A_ mRNA before and after induction of experimental renal fibrosis on control kidneys. RNAscope for ET_A_ mRNA (red) on kidney sections of control kidneys with co-hybridization of PDGFR-β (green), under basal conditions, after 3-week adenine treatment and 5-day UUO. Arrows indicate renal vessels, arrowheads ET_A_ expression in interstitial mesenchymal cells. Scale bars = 50μm

Upregulation of ET_A_-R mRNA in experimental kidney disease mainly occurred in renal interstitial cells which are substantiated by co-hybridization of ET_A_-R and PDGFR-β in renal interstitial fibroblasts that showed an enhanced expression of both genes (Fig. 7). During adenine treatment and UUO, renal ET_B_-R mRNA abundance remained unaltered as already shown in Figure 5C.

The upregulation of ET_A_-R gene expression in the stromal compartment in conjunction with the enhanced endothelial and tubular expression of the ligand ET-1 during experimentally induced kidney fibrosis raised the question about the role of enhanced endothelin signaling in stromal cells for the development of interstitial fibrosis. Since, in addition to the dominant ET_A_-R expression in the FoxD1 compartment, vascular smooth muscle cells show a weak abundance of ET_B_-R, we addressed this question by generating mice with deletions of both endothelin receptors in the stroma cell compartment (ET_A_^flfl^ ET_B_^flfl^ FoxD1^Cre+^ mice).

### Renal function of ET_A_^flfl^ ET_B_^flfl^FoxD1^Cre+^ mice is apparently normal

Endothelin receptor knockout mice developed normally. They had no significant difference in body weight nor showed a different kidney to body weight ratio compared to control littermates (Table 4).

**Table 4:** Kidney developmental parameters under basal conditions in control and ET_A_^flfl^ ET_B_^flfl^ FoxD1^Cre+^ mice. Value are means ± SEM; *n*=11–15 mice

**Kidney developmental parameters**	**ET**_**A**_^**flfl**^**ET**_**B**_^**flf**^	**ET**_**A**_^**flfl**^**ET**_**B**_^**flfl**^**FoxD1** ^**Cre+**^
Body weight (g)	23.6 ± 0.69	23.06 ± 0.90
Two kidney-to-body weight ratio (%)	1.13 ± 0.02	1.11 ± 0.02

Gross renal histology revealed no apparent abnormality in ET-Ko mice. In line, renal functional parameters in ET-Ko mice were not changed compared to controls (Table 5). This suggests that deletion of both endothelin receptors in renal stromal progenitors and their descendants does not disturb normal kidney function.

**Table 5:** Renal functional parameter under basal conditions in control and ET_A_^flfl^ ET_B_^flfl^ FoxD1^Cre+^ mice. Value are means ± SEM; *n*=11–15 mice.

**Renal functional parameters**	**ET**_**A**_^**flfl**^**ET**_**B**_^**flfl**^	**ET**_**A**_^**flfl**^**ET**_**B**_^**flfl**^**FoxD1** ^**Cre+**^
Systolic blood pressure (mmHg)	128.5 ± 1.10	126.4 ± 1.15
**G**lomerular **f**iltration **r**ate/100g bw) (μl/min)	1249.8 ± 170.2	1219.3 ± 230.6
Urine sodium (mmol/l)	129.1 ± 34.7	153.8 ± 63.1
Urine potassium (mmol/l)	232.4 ± 63.5	236.2 ± 45,8
Urine osmolality (mosmol/kg)	1940.0 ± 237.9	1785.0 ± 207.8
Plasma urea concentration (mg/dl)	73.08 ± 1.99	75.91 ± 3.07
Plasma creatinine concentration (mg/dl)	0.75 ± 0.02	0.76 ± 0.02
Hematocrit (%)	54.9 ± 1.4	54.8 ± 0.3
Plasma erythropoietin (pg/ml)	284.7 ± 40.4	316.7 ± 72.7
Plasma renin concentration (ng ANGI/ml*h)	*106.45 ± 14.3*	*80.60 ± 7.11*

### Endothelin system in ET-Ko mice in health and disease

**Fig. 8:**
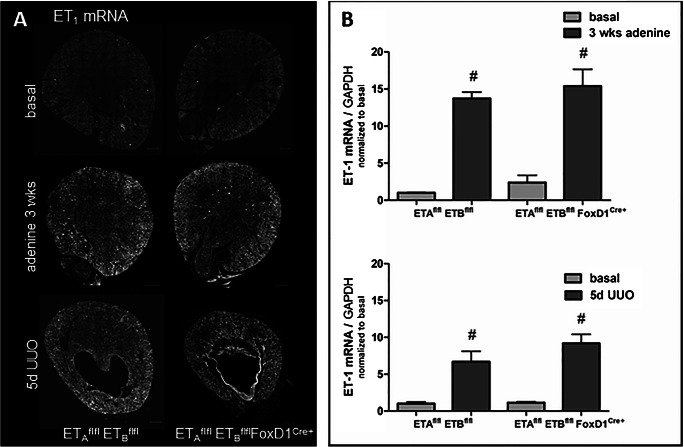
ET-1 mRNA abundance in control and ET-Ko kidneys under basal and pathological conditions. **A** RNAscope showing ET-1 mRNA expression on whole kidney sections of both genotypes under basal conditions, after adenine feeding for 3 weeks and UUO for 5 days. There was no difference between the genotypes under any of the conditions analyzed. Scale bars = 500μm. **B** Expression levels of ET-1 mRNA in adenine-induced nephropathy and after 5-day UUO of control and ET-Ko mice. ET-1 mRNA showed a steep increase after 3-week adenine treatment and 5-day UUO with no difference between genotypes. All data are means ± SEM of 5–8 animals per condition. # is p < 0.05 compared to the respective basal kidneys

In endothelin receptor knockout mice, renal endothelin-1 mRNA expression under basal conditions was similar to control animals. Accordingly, also adenine feeding and UUO led to similar increases of ET-1 mRNA expression in ET-Ko mice as observed in controls (Fig. 8). The comparison of the ET-1 protein expression in healthy and fibrotic kidneys of control and ET-Ko mice yielded a comparable result (Suppl-Fig.1).

**Fig. 9:**
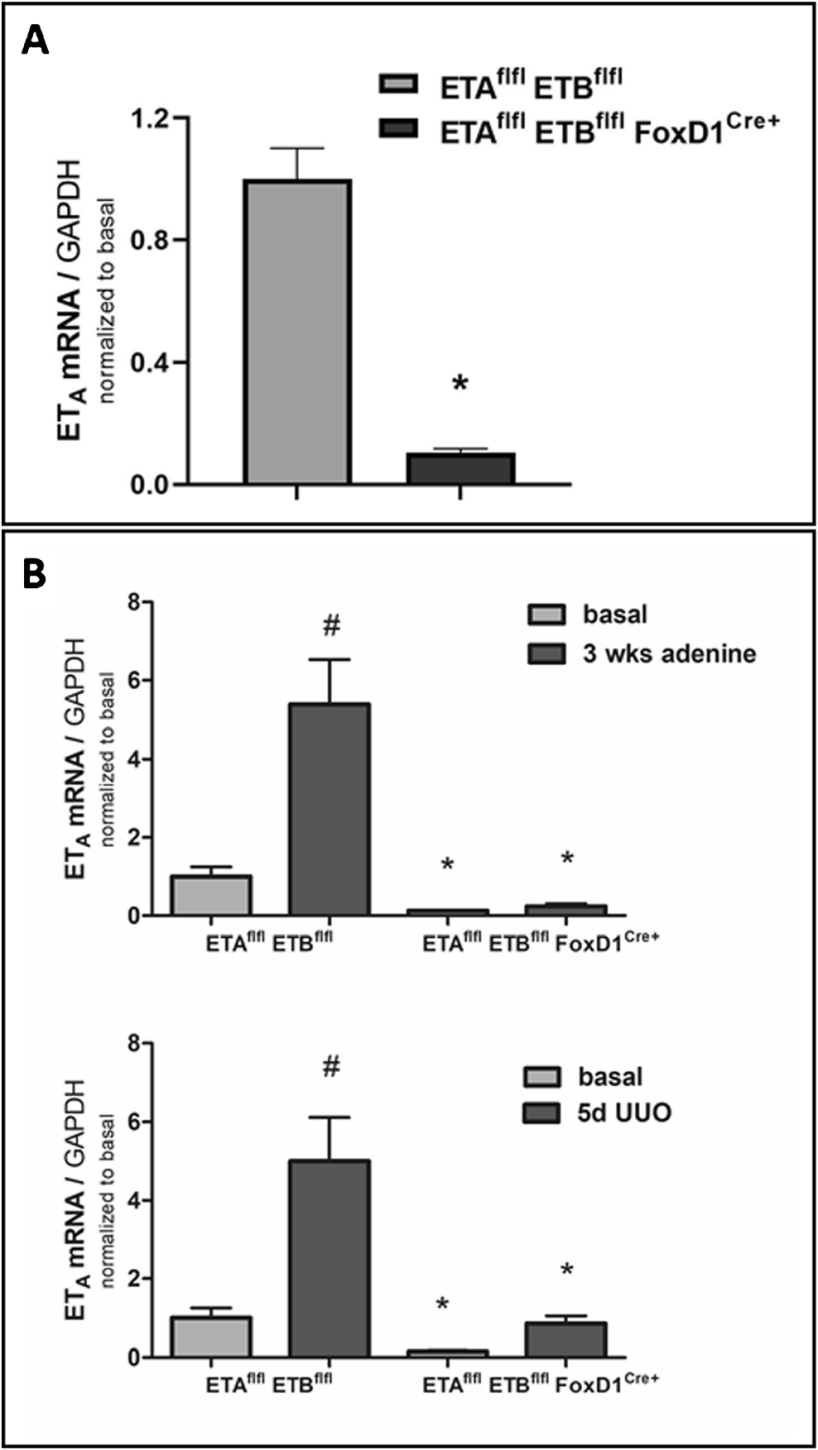
ET_A_ mRNA abundance in control and ET-Ko kidneys under basal and pathological conditions. **A** Expression levels of ET_A_ mRNA in control and ET-Ko mice. ET_A_ mRNA decreased about 90% in knockout mice. All data are means ± SEM of 10–12 mice per condition. Single asterisk is *p*<0.05 compared to untreated controls. **B** Expression levels of ET_A_ mRNA in adenine-induced nephropathy (above) and after 5-day UUO (below) of control and ET-Ko mice. ET_A_ mRNA showed a steep increase in kidneys of controls in both experimental models. In ET-Ko mice, ET_A_ mRNA was not upregulated after adenine treatment and 5-day UUO. All data are means ± SEM of 5–8 animals per condition. Single asterisk is *p* < 0.05 compared to the respective controls. Number sign is *p* < 0.05 compared to the respective basal kidneys

Basal ET_A_ mRNA expression in endothelin receptor knockout kidneys was significantly reduced by around 90% compared to the kidneys of control mice. (Fig. 9A). The marked increase in ET_A_ mRNA observed in control kidneys during adenine feeding or UUO was also greatly attenuated in the ET-Ko kidneys (Fig. 9B), which suggests that the increase in ET_A_-R gene expression during the experimental fibrosis probably took place mainly in the stromal FoxD1 compartment.

**Fig. 10:**
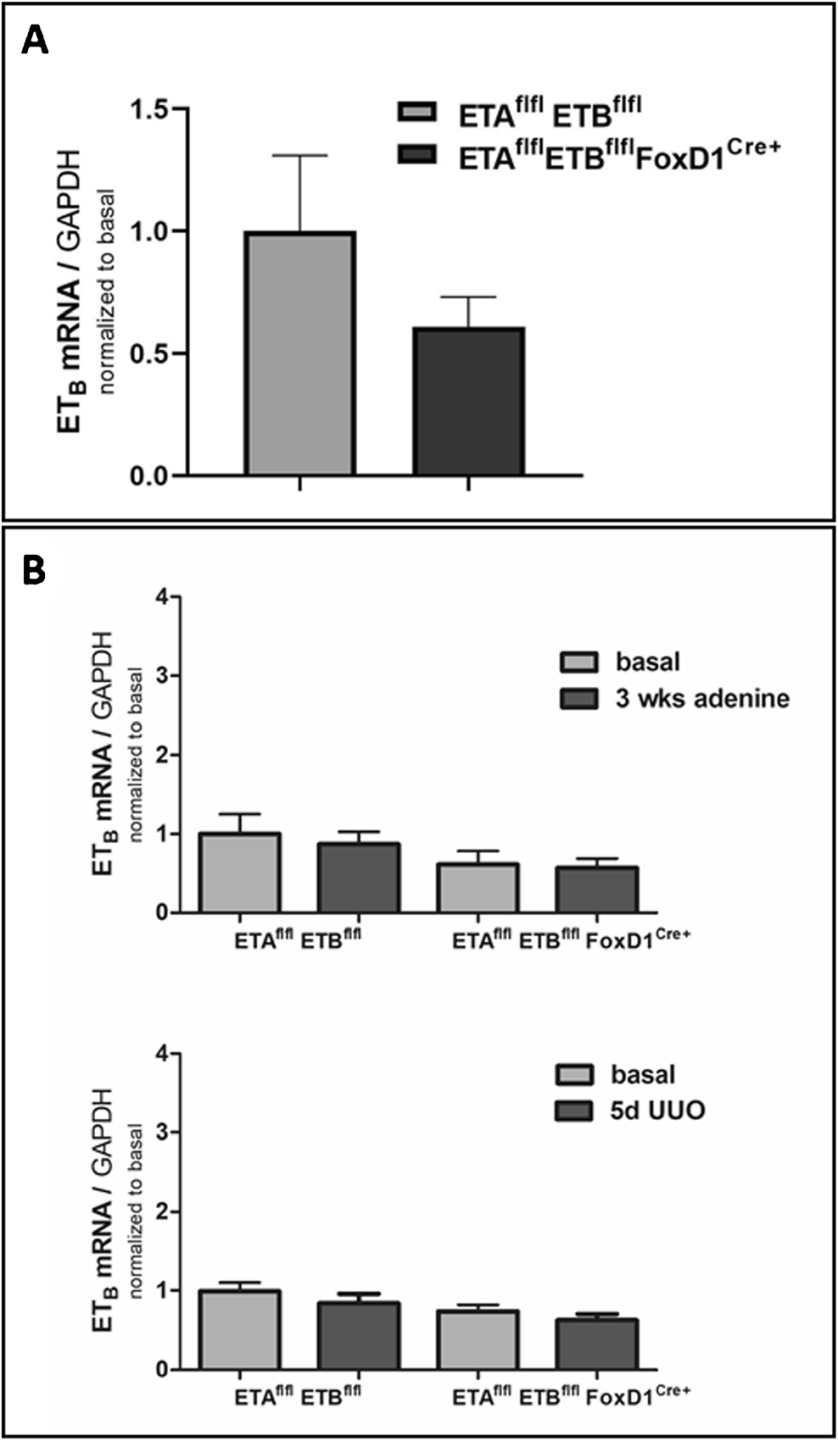
ET_B_ mRNA abundance in control and ET-Ko kidneys under basal and pathological conditions. **A** Expression levels of ET_B_ mRNA in control and ET-Ko mice. ET_B_ mRNA decreased about 25% in knockout mice. All data are means ± SEM of 10–12 mice per condition. **B** Expression levels of ET_B_ mRNA in adenine-induced nephropathy (above) and after 5-day UUO (below) of control and ET-Ko mice. ET_B_ mRNA remained unchanged in both experimental models. All data are means ± SEM of 5–8 animals per condition

The basal ET_B_ mRNA abundance was reduced by about 25% in the endothelin receptor knockout kidneys (Fig. 10A), indicating that ET_B_ is mainly expressed outside the FoxD1 compartment with the exception of its expression in vascular smooth muscle cells. This could explain the moderate decrease in its expression in the knockout model. Adenine treatment and UUO did not change the ET_B_-R mRNA expression in control and endothelin receptor knockout kidneys (Fig. 10B).

### Disruption of the ET signaling in stromal cells does not influence myofibroblast development in experimental kidney disease

**Fig. 11:**
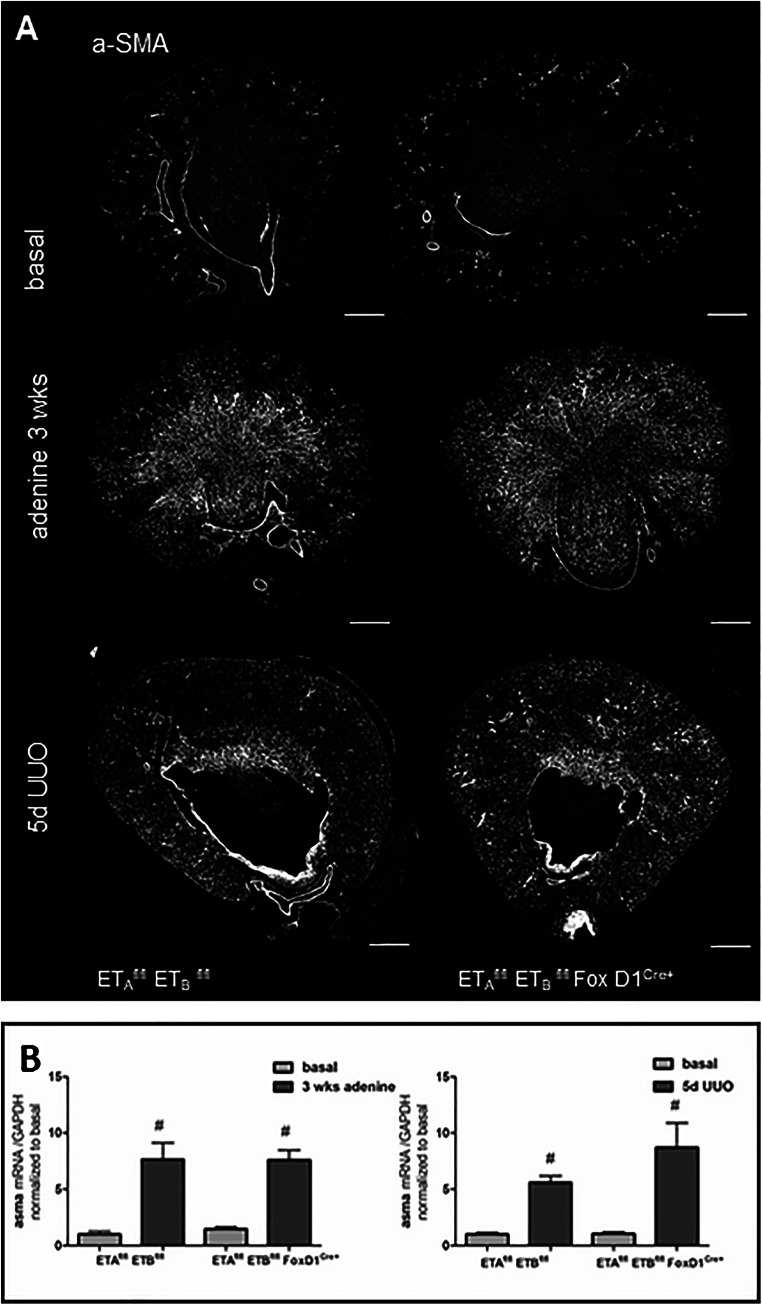
α-SMA mRNA abundance in control and ET-Ko mice under basal and pathological conditions. **A** RNAscope showing α-SMA mRNA on whole kidney sections of both genotypes under basal conditions, after adenine feeding for 3 weeks and UUO for 5 days. Scale bars = 500μm. **B** Expression levels of α-SMA mRNA in adenine-induced nephropathy (left) and after 5-day UUO (right) of control and ET-Ko mice. α-SMA mRNA showed a steep increase in the kidneys of control mice in both experimental models without difference between genotypes. All data are means ± SEM of 5–8 animals per condition. Number sign is *p* < 0.05 compared to the respective basal kidneys

Development of fibrosis is typically characterized by the formation of myofibroblasts that express α-SMA. In the healthy kidney, expression of α-SMA mRNA was mainly seen in the medial layer of renal vessels (Fig. 11A). Adenine treatment and UUO for 5 days led to a strong upregulation of α-SMA expression (Fig. 11) and appeared in interstitial, fibroblast-like cells mainly in the outer and inner medulla of the kidney, whereas no significant difference could be observed between control and knockout mice. (Fig 11A,B). Immunohistochemical analysis of α-SMA protein expression in basal and fibrotic kidneys of the two genotypes gave the same result (Suppl.Fig.2).

### Disruption of the ET signaling in stromal cells does not influence profibrotic gene expression in experimental kidney disease

**Fig. 12:**
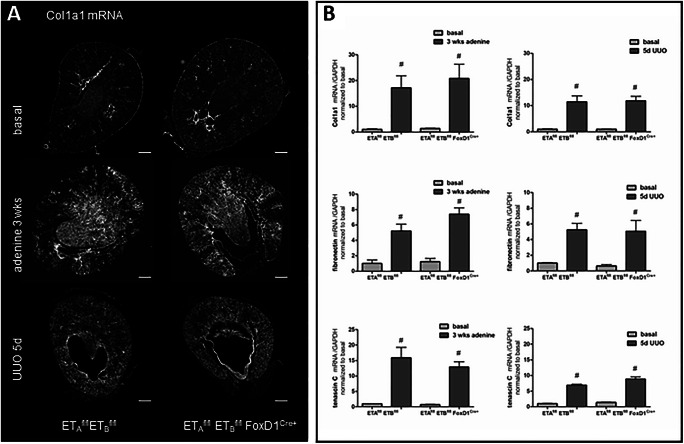
mRNA abundance of profibrotic markers in control and ET-Ko mice under basal and pathological conditions. **A** RNAscope for Col1a1 mRNA on whole kidney sections of both genotypes under basal conditions, after adenine feeding for 3 weeks and UUO for 5 days. Scale bars = 500μm. **B** Expression levels of the fibrotic marker Col1a1, fibronectin, and tenascin C mRNA in adenine-induced nephropathy and after 5-day UUO of both genotypes. All markers showed a steep increase in the kidneys of both genotypes for each experimental model. All data are means ± SEM of 5–8 animals per condition. Number sign is *p* < 0.05 compared to the respective basal kidneys

To examine a potential role of ET_A_-R and ET_B_-R expression in FoxD1-derived cells for deposition of extracellular matrix, we compared the expression of the key fibrotic marker collagen1a1 (Col1a1), fibronectin, and tenascin C between control and ET-Ko mice. Experimental renal fibrosis led to 20- and 10-fold upregulation of Col1a1 mRNA with no significant differences between genotypes (Fig. 12). Again, the analysis of the protein expression by immunohistochemistry showed no differences in Col1a1 expression between the two genotypes (Suppl. Fig.3).

Adenine feeding and UUO also led to strong increases of fibronectin and tenascin C mRNA expressions which were also not different between control and endothelin receptor knockout mice (Fig. 12B).

### Disruption of the ET signaling in stromal cells does not affect proinflammatory gene expression in experimental kidney disease

**Fig. 13:**
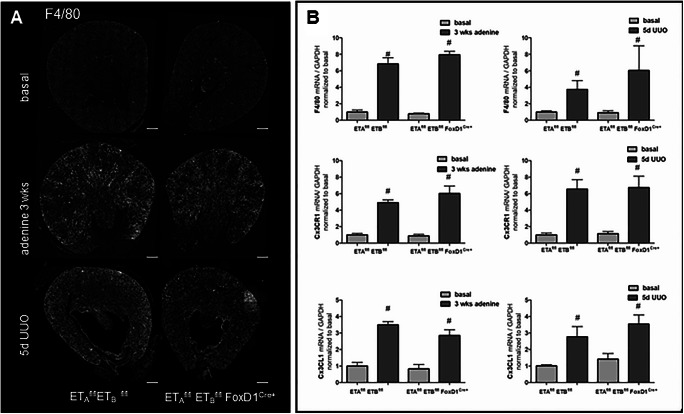
mRNA abundance of proinflammatory markers in control and ET-Ko mice under basal and pathological conditions. **A** RNAscope for F4/80 mRNA on whole kidney sections of both genotypes under basal conditions, after adenine feeding for 3 weeks and UUO for 5 days. Scale bars = 500μm. **B** Expression levels of F4/80, Cx3CR1, and Cx3CL1 mRNA in adenine-induced nephropathy and after 5-day UUO of both genotypes. All markers showed a steep increase in kidneys of both genotypes for each experimental model. All data are means ± SEM of 5–8 animals per condition. Number sign is *p* < 0.05 compared to the respective basal kidneys

The influx of monocytes/macrophages and lymphocytes into the kidney in states of experimental renal disease leads to chronic interstitial inflammation and subsequent interstitial fibrosis [60, 66] *.* We evaluated macrophage infiltration in fibrotic kidneys by analysis of F4/80 expression using RNAscope and real-time PCR. F4/80 is a well-known and widely used marker of murine macrophage populations. Both adenine treatment and UUO markedly elevated F4/80 mRNA expression in control and ET-Ko mice without any difference between genotypes (Fig. 13A,B). Additionally, we studied the expression of the chemokine fractalkine (Cx3CL1) and its receptor Cx3CR1 as a marker for inflammation which again did not show any difference between control and ET-Ko mice (Fig. 13B).

### Disruption of the ET signaling in stromal cells does not affect urinary ET-1 and albumin secretion in experimental kidney disease

Urinary ET-1 excretion showed no difference between controls and ET-Ko mice under basal conditions. Adenine treatment increased ET-1 excretion 2-fold in controls and 1.8-fold in ET-Ko mice, whereas UUO does not lead to a significant increase in ET-1 concentrations in both genotypes (Table 6). No difference was observed between controls and ET-Ko mice with regard to urinary albumin excretion. Furthermore, neither adenine treatment nor UUO led to an increase in albumin excretion (Table 6).

**Table 6:** Urinary ET-1 and albumin concentration in control and ET_A_^flfl^ ET_B_^flfl^ FoxD1^Cre+^ mice after adenine treatment and 5d UUO. Value are means ± SEM; n=4-7 mice. Single asterisk is p < 0.05 compared to the respective controls

	***ET***_***A***_^***flfl***^ ***ET***_***B***_^***flfl***^			***ET***_***A***_^***flfl***^ ***ET***_***B***_^***flfl***^ ***FoxD1*** ^***Cre+***^		
	***basal***	***3 wks adenine***	***5-d UUO***	***Basal***	***3 wks adenine***	***5-d UUO***
ET-1 (pg/ml)	0.66 ± 0.07	1.37 ± 0.19*	0.77 ± 0.11	1.00 ± 0.18	1.79 ± 0.29*	1.14 ± 0.18
albumin (μg/ml)	14.52 ± 1.45	12.47 ± 1.71	11.16 ± 1.96	12.59 ± 1.67	16.03 ± 2.46	10.97 ± 1.82

## Discussion

The aim of this study was to clarify the role of ET-1 signaling in stromal cells for the progression of renal fibrosis in two models of experimental renal disease. We found that adenine-induced nephropathy and unilateral ureter occlusion led to an upregulation of mainly tubular ET-1 expression and to an upregulation of ET_A_ gene expression in the stromal cell compartment which includes also interstitial cells. Genetic ablation of endothelin receptors from the stromal cell compartment, however, did not change the upregulated expressions of profibrotic and proinflammatory markers during experimentally induced kidney fibrosis.

Our findings of an activation of the endothelin system in fibrotic kidney disease is in accordance with previous reports, which demonstrated either an enhanced ET-1 gene expression [1, 16, 43, 44] or an increased ET_A_ gene expression [9, 16] in experimentally induced kidney fibrosis. We now extend these findings by showing the localization of increased ET-1 and ET_A_ gene expression. Our data suggest that the enhanced expression of ET-1 mainly occurs in tubuli, while the expression of ET_A_ almost exclusively occurs in the stromal cell compartment , which includes vascular smooth muscle cells, renin producing cells, mesangial cells, and resident interstitial cells. From these findings, we conclude that in states of kidney fibrosis, endothelin signaling in the stromal cell compartment and also in interstitial cells was enhanced. Our findings further show that genetic constitutive deletion of ET-R from the stroma cell population did not change the characteristic increases of profibrotic and proinflammatory gene expression during fibrotic disease, suggesting that endothelin signaling in stromal cells has less impact for the development of kidney fibrosis. On the first glance, this finding contrasts with a number of reports suggesting a profibrotic and proinflammatory role of endothelin during kidney disease. Our findings, moreover, appear to be in contrast with studies showing an attenuating effect of ET_A_ antagonists in diabetes-related kidney damage [13, 23, 53, 58]. Since in these latter studies endothelin antagonists were systemically administered and since the patho-mechanisms of diabetes-related kidney fibrosis may differ from those of tubulointerstitial fibrosis as examined in this study, the comparability of our results with those of the aforementioned studies is limited.

In this context, clinical trials should also be mentioned that show the therapeutic potential of ET_A_ antagonists in kidney diseases and provide data that contradict our findings.

These trials performed with various ETA antagonists show reno-protective effects by reducing proteinuria in patients with chronic kidney disease and type 2 diabetes [12, 21, 22, 34, 36, 42, 54, 61] which shows us that the results from a selective, cell-specific deletion of the ET receptors in the animal model are hardly transferable to human kidney diseases.

An obvious explanation of the divergent findings could be a relevant role of the ET_B_ for kidney fibrosis, which we mainly localized in endothelial and tubular cells, what is in good accordance with previous findings [3, 38, 49, 67]. Although the expression of ET_B_ mRNA did not change during kidney fibrosis, the increased expression of ET-1 mRNA in tubuli and endothelial cells could lead to an activation of endothelin signaling through ET_B_, because ET-1 is known to exert para- and autocrine effects. Indications to the relevance of ET_B_ in fibrosis came from studies in which ET_B_-specific antagonists prevented renal damage in experimental models of renal fibrosis [56]. It is conceivable therefore that tubular ET_B_ signaling initiates or contributes to renal fibrosis such as epithelial to mesenchymal transformation [8, 56, 59, 68]. Increased activation of ET_B_-R in tubuli or endothelial cells could also induce the production and release of cytokines, such as TGFß1, that induce matrix production in interstitial cells [4, 19, 47], or cytokines, like Cx3CL1 attracting inflammatory cell [69].

An interesting point to mention is that overexpression of ET-1 leads to inflammation of the kidney. Hocher et al. [24] showed an increase in iNOS expression and an infiltration of CD4-positive lymphocytes and macrophages in the kidneys of ET-1 transgenic mice with overexpression of ET-1. Several studies have confirmed that renal inflammation is closely related to the formation of fibrosis, and it is assumed that macrophages promote inflammation in the early stages of kidney damage [17]. Therefore, when interpreting our results, we must also consider a connection between inflammation and fibrosis.

Another interesting aspect is provided by a work of Tsuprykov et al. in which ET-1 even shows antifibrotic effects in renal interstitial fibrosis and glomerulosclerosis [62]. This work shows that in eNOS -/- mice that develop renal interstitial and glomerular damage, the increase in expression of genes involved in renal fibrosis is markedly reduced by overexpression of ET-1.

Certainly, we cannot exclude that a minor residual expression of ET-R was sufficient to maintain an enhanced endothelin signaling in stromal cells, because Cre-lox recombination does normally not produce complete gene disruptions. However, in view of the marked changes of ET_A_ mRNA in combination with unaffected mRNAs for profibrotic and proinflammatory markers, we consider this scenario as a less likely explanation.

Our data show the activation of the ET-system during the development of kidney fibrosis that includes an upregulation of ET-1 synthesis in endothelial and tubular cells but also the enhanced expression of ET_A_ in FoxD1-derived mesenchymal progenitor cell population. Our findings further demonstrate that genetic deletion of ET-R in this compartment had no effect on development and progression of renal fibrosis. We now suspect that cellular processes other than the activation of fibroblasts play an essential role in renal fibrosis.

## Supplementary information

Suppl. Figure 1: ET-1 protein abundance in control and ET-Ko mice under basal and pathological conditions. Immunohistochemical analysis showing ET-1 staining on kidneys sections of both genotypes under basal conditions, after adenine feeding for 3 weeks and UUO for 5 days. In order to make the localization of the Col1a1 signals (red) clear, the kidney morphology was highlighted with an uncolored, turquoise channel. Scale bars = 200μm. (PNG 11675 kb)

High Resolution Image (TIF 2533 kb)

Suppl. Figure 2: α -SMA protein abundance in control and ET-Ko mice under basal and pathological conditions. Immunohistochemical analysis showing α -SMA staining on whole kidney sections of both genotypes under basal conditions, after adenine feeding for 3 weeks and UUO for 5 days. In order to make the localization of the Col1a1 signals (red) clear, the kidney morphology was highlighted with an uncolored, turquoise channel. Scale bars = 500μm. (PNG 2778 kb)

High Resolution Image (TIF 634 kb)

Suppl. Figure 3: Col1a1 protein abundance in control and ET-Ko mice under basal and pathological conditions. Immunohistochemical analysis showing Col1a1 staining on whole kidney sections of both genotypes under basal conditions, after adenine feeding for 3 weeks and UUO for 5 days. In order to make the localization of the Col1a1 signals (red) clear, the kidney morphology was highlighted with an uncolored, turquoise channel. Scale bars = 500μm. (PNG 2957 kb)

High Resolution Image (TIF 662 kb)
