# Supplementary figures and images for: Endothelin receptors in renal interstitial cells do not contribute to the development of fibrosis during experimental kidney disease
Source: Pflugers Arch. 2021 Aug 6;473(10):1667–83. doi: 10.1007/s00424-021-02604-4 (PMC8433107; doi:10.1007/s00424-021-02604-4)

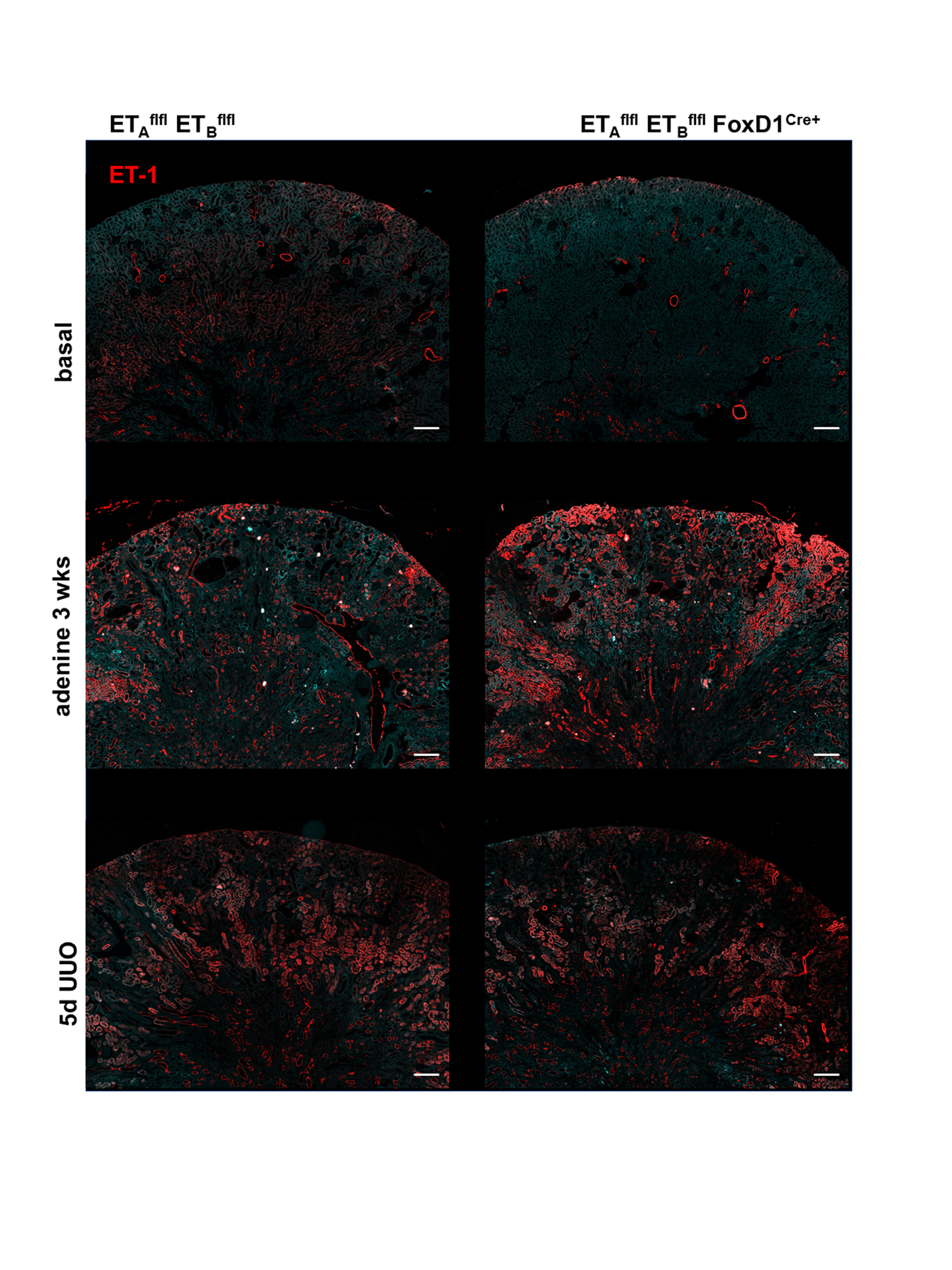

Supplement: Supplementary file 1 — ET-1 protein abundance in control and ET-Ko mice under basal and pathological conditions. Immunohistochemical analysis showing ET-1 staining on kidneys sections of both genotypes under basal conditions, after adenine feeding for 3 weeks and UUO for 5 days. In order to make the localization of the Col1a1 signals (red) clear, the kidney morphology was highlighted with an uncolored, turquoise channel. Scale bars = 200μm. (PNG 11675 kb) [file 424_2021_2604_Fig14_ESM.png]

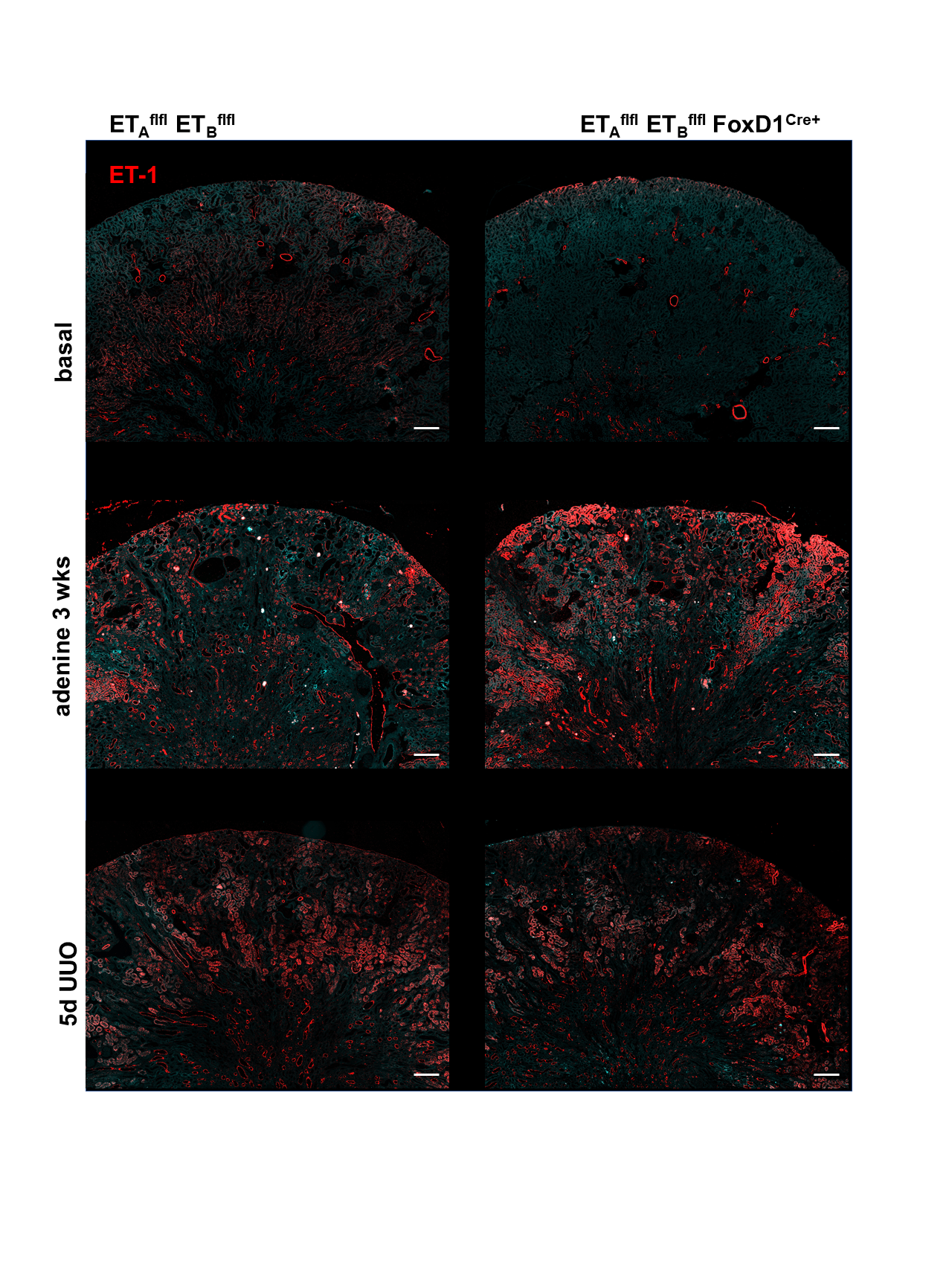

Supplement: Supplementary file 2 — High Resolution Image (TIF 2533 kb) [file 424_2021_2604_MOESM1_ESM.tif]

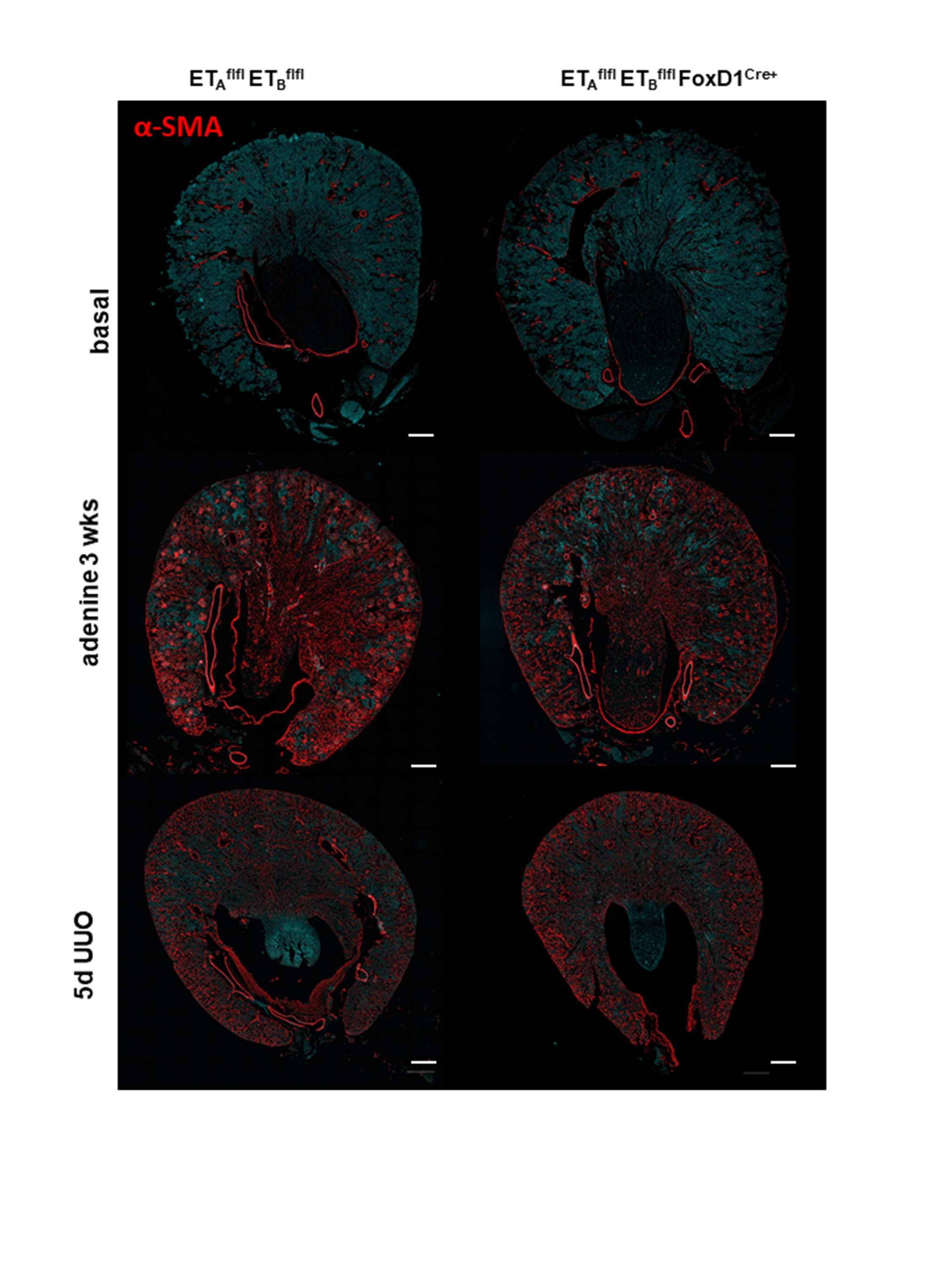

Supplement: Supplementary file 3 — α -SMA protein abundance in control and ET-Ko mice under basal and pathological conditions. Immunohistochemical analysis showing α -SMA staining on whole kidney sections of both genotypes under basal conditions, after adenine feeding for 3 weeks and UUO for 5 days. In order to make the localization of the Col1a1 signals (red) clear, the kidney morphology was highlighted with an uncolored, turquoise channel. Scale bars = 500μm. (PNG 2778 kb) [file 424_2021_2604_Fig15_ESM.png]

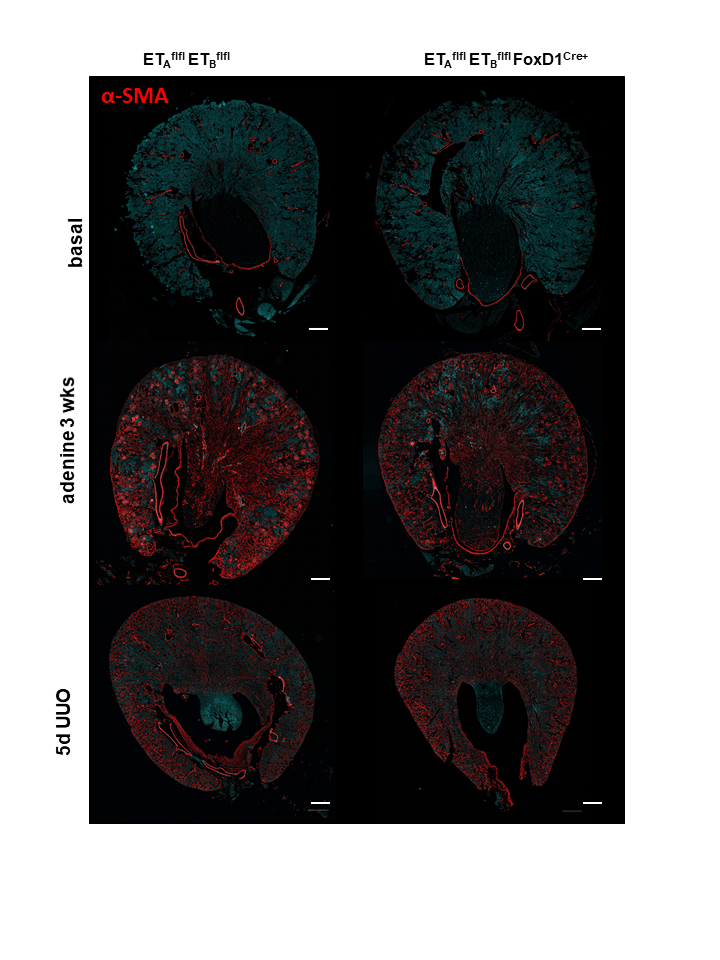

Supplement: Supplementary file 4 — High Resolution Image (TIF 634 kb) [file 424_2021_2604_MOESM2_ESM.tif]

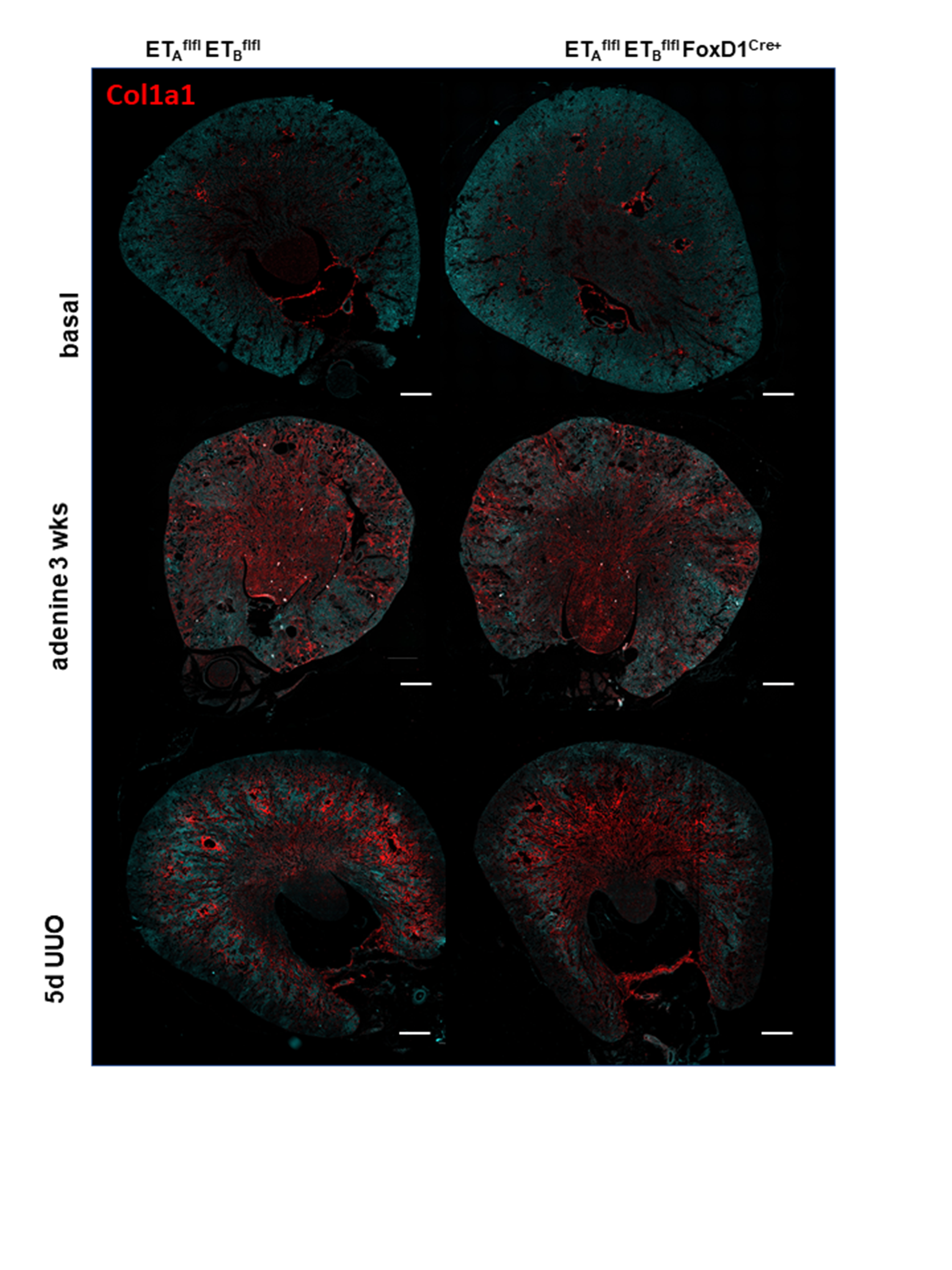

Supplement: Supplementary file 5 — Col1a1 protein abundance in control and ET-Ko mice under basal and pathological conditions. Immunohistochemical analysis showing Col1a1 staining on whole kidney sections of both genotypes under basal conditions, after adenine feeding for 3 weeks and UUO for 5 days. In order to make the localization of the Col1a1 signals (red) clear, the kidney morphology was highlighted with an uncolored, turquoise channel. Scale bars = 500μm. (PNG 2957 kb) [file 424_2021_2604_Fig16_ESM.png]

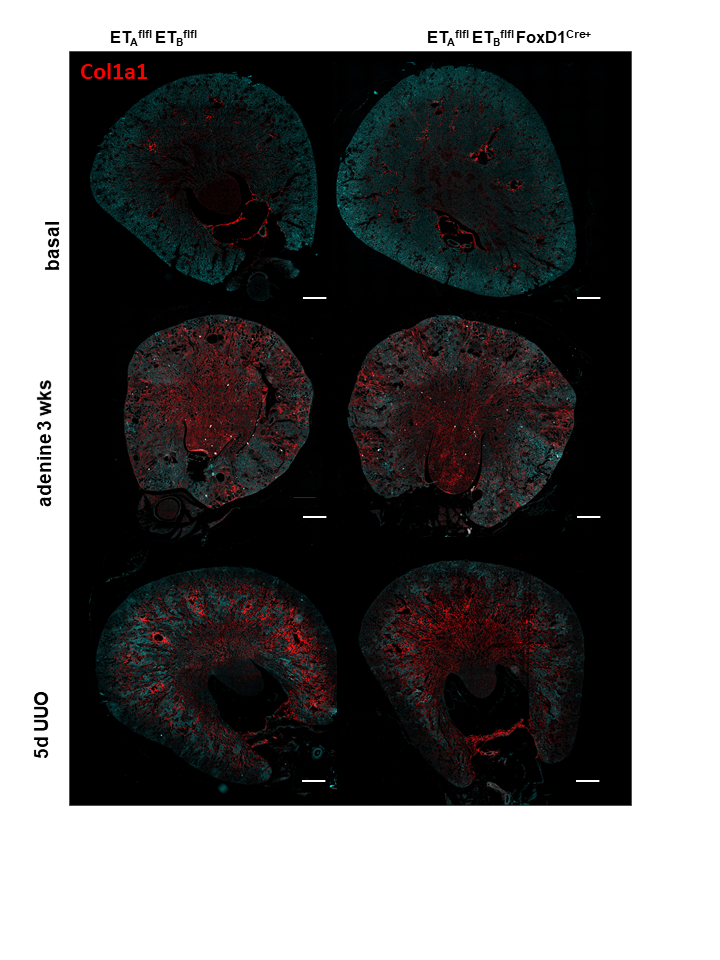

Supplement: Supplementary file 6 — High Resolution Image (TIF 662 kb) [file 424_2021_2604_MOESM3_ESM.tif]
